# Trait Anxiety Has Effect on Decision Making under Ambiguity but Not Decision Making under Risk

**DOI:** 10.1371/journal.pone.0127189

**Published:** 2015-05-22

**Authors:** Long Zhang, Kai Wang, Chunyan Zhu, Fengqiong Yu, Xingui Chen

**Affiliations:** 1 Department of Neurology, The First Affiliated Hospital of Anhui Medical University, Hefei, China; 2 Laboratory of Neuropsychology, Anhui Medical University, Hefei, China; Radboud University Nijmegen, NETHERLANDS

## Abstract

Previous studies have reported that trait anxiety (TA) affects decision making. However, results remain largely inconsistent across studies. The aim of the current study was to further address the interaction between TA and decision making. 304 subjects without depression from a sample consisting of 642 participants were grouped into high TA (HTA), medium TA (MTA) and low TA (LTA) groups based on their TA scores from State Trait Anxiety Inventory. All subjects were assessed with the Iowa Gambling Task (IGT) that measures decision making under ambiguity and the Game of Dice Task (GDT) that measures decision making under risk. While the HTA and LTA groups performed worse on the IGT compared to the MTA group, performances on the GDT between the three groups did not differ. Furthermore, the LTA and HTA groups showed different individual deck level preferences in the IGT: the former showed a preference for deck B indicating that these subjects focused more on the magnitude of rewards, and the latter showed a preference for deck A indicating significant decision making impairment. Our findings suggest that trait anxiety has effect on decision making under ambiguity but not decision making under risk and different levels of trait anxiety related differently to individual deck level preferences in the IGT.

## Introduction

In recent years, researchers have accepted the hypothesis that emotions play an important role in human cognition, and many investigators have paid extensive attention to the issue of how emotions influence higher cognitive processes [1]. Anxiety is a crucial emotion in our daily life. According to the evolutionary perspective, anxiety is also an adaptive emotion and has a survival value by enabling the quick and accurate detection of a potential threatening stimulus or situation. However, inappropriate activation of the anxiety system may result in anxiety disorder or other psychiatric disorders [2]. The progression from normal to pathological forms of anxiety is thought to be a continuum. People with a high disposition for anxiety, i.e., high trait anxiety (TA), are at more risk for developing one or more anxiety disorders [3,4]. Trait anxiety reflects individual differences in sensitivity to negative or threating stimulus. These individual differences are expressed in attentional, memory and interpretative biases toward aversive stimuli, reflecting changes to the processing of environmental information [5,6]. Many studies have investigated the effects of trait anxiety on cognitive performances: high trait anxiety individuals show evidence for impaired inhibitory learning of the threat cue [7], display impaired performance on prefrontal-dependent cognitive control tasks [8] and are associated with reduced representations capacity in working memory [9].

Recent studies have suggested that variation in trait anxiety can also result in individual differences in emotional reactivity, with direct results on decision making [3,10,11]. Decision making is a complex process that includes measuring and weighing short-term and long-term costs and gains of different options. The decision making process is a dynamic interaction between an impulsive system, which responds to immediate outcomes, and a reflective system, which controls long-term outcomes, ultimately integrating into a profitable choice strategy [12]. There are at least two types of decision making, which differ in mainly the degree of uncertainty and how much useful information about consequences and their probabilities provided to decision maker [13]. In some situations, outcomes and probabilities are implicit and the decision makers must access effective information by means of processing feedback of previous choices. This decision making is often termed decision making under ambiguity. Investigators usually use the Iowa Gambling Task (IGT) [14] to imitate the decision making of real life by constraining different rewards, punishments and many uncertainties, to estimate decision making under ambiguity. In the IGT, participants have to maximize a fictitious amount of money by successively choosing cards from four different card decks that differ in the frequency and magnitude of monetary gains and losses. Participants do not know the amount of cards they need to choose and also which card decks are advantageous (i.e. coupling with high gains but even higher losses and leading to a positive overall balance in the long term) or disadvantageous (i.e. coupling with small gains but even smaller losses and leading to a negative overall balance in the long term). Therefore, the possible choices are full of ambiguity and participants have to learn to avoid the disadvantageous card decks using the feedback from previous trials.

In contrast to decision making under ambiguity, some decision situations contain explicit information about the potential consequences of the choices and their probabilities are provided. This type of decision making is referred to as decision making under risk. In the laboratory environment, decision making under risk is usually measured by the Game of Dice Task (GDT) [15], in which explicit rules for gains, losses and probabilities are available from the beginning of the task. In the GDT, subjects are required to choose among four different options to maximize fictitious starting money, and the different options are explicitly related to a specific amount of gain/loss. Some options, which are related with high potential gains/losses but low winning probabilities are risky; and other options, which are related with lower potential gains/losses but higher winning probabilities are non-risky. Thus, subjects can estimate the risk related with each option and may apply strategies to maximize profit.

Neuropsychological and neuroimaging studies have identified that a number of brain areas, such as ventromedial prefrontal cortex (vmPFC)/orbitofrontal prefrontal cortex (OFC), amygdala, dorsolateral prefrontal cortex (dlPFC) and anterior cingulate cortex, involve in decision making [16,17,18]. Unimpaired IGT performance has associated with intact function of the OFC [17,18,19]. Even in a rat analogue of the IGT, rats with OFC lesions preferred to choose larger but more unpredictable rewards over smaller but more reliable rewards under conditions of uncertainty and ambiguity [20,21] or showed inadapted behaviors and impaired performance [22,23]. However, previous studies have suggested that the dlPFC plays a major role in the GDT. Neuropsychological studies have found that subjects with compromised dlPFC function show impaired performance on the GDT [24,25]. Neuroimaging studies have demonstrated that decision making under risk as assessed by the GDT depends on the activation of the dlPFC [26]. Several brain regions linked to decision making also play an important part in anxiety. For example, some neuroimaging studies have showed that trait anxiety is connected with increased activation of amygdala and vmPFC/OFC [27,28], reduction of cortical thickness in OFC [4] and altered activity of the dlPFC [29]. Therefore, trait anxiety may affect decision making under ambiguity and decision making under risk.

To our best knowledge, however, there has been no study to examine decision making under ambiguity and decision making under risk in the same group of healthy participants with different levels of TA. Here we therefore classified healthy participants on the Trait Anxiety Inventory (TA-I, a section of the widely used State-Trait Anxiety Inventory) [30] and related their TA scores to their performances on decision making under ambiguity as measured by the IGT and decision making under risk as measured by the GDT to demonstrate interactions between TA and decision making. To control for differences in executive functions that are related with performance on the GDT [31,32], participants performed the Wisconsin Card Sorting Test [33] and the Trail Making Test [34]. The Wisconsin Card Sorting Test was used in the study by de Visser et al. [3] as well. Since depressed individuals exhibit abnormal behavioral responses to rewards and punishments and have difficulty with decision making [35,36], the Beck Depression Inventory II (BDI-II) [37] was used to measure severity of depression.

## Methods

### Participants

A sample consisting of 642 participants (332 males, 310 females) was initially enrolled in the study at the Anhui Medical University campus (age: *M* = 19.17, *SD* = 1.29). The vast majority of participants were 18 years old or older. There were 30 participants, less than 10%, who were under the age of 18. We explained the background and significance of our study in class, and they gave their written informed consent to participate. For participants under the age of eighteen, we obtained written informed consent by post from their parents on behalf of the students enrolled in our study. Each participant received ¥10 for participating. The study and the consent procedure were approved by the Ethics Committee of Anhui Medical University.

All participants were assessed with a questionnaire. The exclusion criteria were current or past diagnosis of psychotic disorder, neurological illness, head injury, drug or alcohol abuse, serious medical illness, or present use of medication. In particular, participants were excluded from the present study if they had any history of depression or had a BDI-II score > 18. We also did not include subjects taking oral contraceptives, though the use of oral contraceptives was found to have no effect on IGT performance [38]. In the end, twelve participants were excluded: two had a history of depression, seven had a BDI-II score > 18 and three were taking medication.

We administered the TA-I to all participants first. Participants were told that they were participating in a psychological study and that they had the right to withdraw from participation at any time. They were also told that there were no right or wrong responses to the questions. Subjects were asked to include their student number and contact information after completing the questionnaire. One week later, the participants belonging to the three groups participated in the decision making tests. They were told that they were randomly selected to take the decision making tests. The one-week time lag allowed researchers enough time to analyze the TA scores for dispositional status of individual differences. The operating procedures of the IGT and GDT were explained in detail and so that they were easy to follow, and all participants indicated that they fully understood the two tasks.

### Trait Anxiety Inventory

The TA-I has 20 items that measure TA, which concerns how the participants feel in general, and their habitual or dispositional feeling of anxiety. Answers to the questionnaire are rated on a 4-point Likert scale ranging from 1 to 4. The sum score of the scale ranges from 20 to 80. Higher scores are associated with higher levels of anxiety. We used the Chinese version of the TA-I [39], which has good reliability and validity. Cronbach’s alpha coefficient for the T-AI was 0.81 in the present study.

### Beck Depression Inventory II

The Beck Depression Inventory II was used to measure severity of depression [37]. It has been used for clinical screening and in epidemiological research. The BDI-II consists of 21 items, which are scored on a 4-point Likert scale (0–3) indicating the severity or frequency of a particular depressive symptom. Scores range 0–63, with higher scores indicating more severe symptoms. We used the Chinese version of the BDI-II, which has been validated for use in the Chinese population and has good reliability and validity [40].

### Decision making under ambiguity

The computerized version of the IGT was used to measure decision making under ambiguity [14,16]. In this task, subjects are instructed to choose one card from four decks of cards (A, B, C and D). After each card selection, they win or lose a specified amount of money. On the IGT, decks A and B yield an average gain of €100 per selection, and decks C and D yield an average gain of €50 per selection. Subjects also encounter losses. 10 selections from decks A or B lead to a net loss of €250, whereas ten selections from decks C or D lead to a net gain of €250. In short, A and B are disadvantageous decks, they include high immediate gains, but even higher losses, resulting in a negative outcome over the long run; decks C and D are advantageous, they produce small immediate gains, but even smaller losses, resulting in a positive outcome in the long term. Moreover, there are also other inequalities between the four decks. For instance, although decks A and B lead to long-term negative outcomes, selections from deck A are punished on 50% of trials but deck B selections are punished on 10% of trials. The immediate losses on deck A are also smaller than those in deck B. Similar differences are seen between decks C (50% losses) and D (10% losses), and the immediate losses on deck C are also smaller than those in deck D [41].

Subjects are told that some decks are better than other decks and they can select cards from any deck. They are told to win as much money as possible with a starting capital over 100 trials. The gain or the loss after each selection, and the new monetary total are shown on the screen. No other information was given. We calculated the total netscore by subtracting the number of disadvantageous choices from the number of advantageous choices to analyze task performance. The 100 trials were divided into five equal blocks, and the netscore of each block of 20 cards was calculated to investigate whether decision making changed during the task. Furthermore, the number of cards selected in individual deck A, B, C and D were calculated to examine individual deck level preference.

### Decision making under risk

To assess decision making under risk, we used the computerized GDT [15]. In the task, subjects roll a virtual die 18 times, with the goal of maximizing their gains with a fictitious starting capital (€1000) by choosing one of four different options. Subjects guess the result of the game and choose to bet on either a single die or one die out of two, three or four dice combinations. They win some money if the chosen number or one of the chosen numbers is thrown, otherwise they lose the same amount of money. Each option is associated with defined gain/loss and different winning probabilities: 1000€ gain/loss with a winning probability of 1:6 for a single number; 500€ gain/loss with a winning probability of 2:6 for combination of two numbers; 200€ gain/loss with a winning probability of 3:6 for combination of three numbers; 100€ gain/loss with a winning probability of 4:6 for combination of four numbers. If, for instance, a participant bets on the combination “one”, “two” and “three”, and a one, two, or three is thrown, the participant wins 200€; however, if a four, five or six is thrown, 200€ are lost. The two former options, which have lower winning probabilities are grouped into risky decisions; the two latter options, which have higher winning probabilities are grouped into non-risky decisions. Additionally, the gain or the loss, the change in capital, and the number of the rest of die throws were presented on the screen after each selection.

For analysis, we calculated a netscore (the number of non-risky choices minus the number of risky choices) to analyze task performance. We also calculated how often the four different options were chosen.

### Neuropsychological background testing

#### Trail Making Test

All participants had completed the Trail Making Test (TMT): Test A and Test B [34]. For Test A, participants were asked to connect 25 encircled numbers, which were distributed on a piece of paper, as accurately and quickly as possible in ascending order. For Test B, participants were asked to connect numbers and letters alternately (e.g., 1, A, 2, B, 3, C, etc.). If a mistake was made, the participant could return to the “circle” where the mistake originated and continue. Test A measures mental tracking and motor speed, and Test B captures selective attention and cognitive flexibility. The amount of time required to complete each test represents the score on each test.

#### Wisconsin Card Sorting Task

Participants in the three groups had also completed the Wisconsin Card Sorting Task (WCST), which measures executive function. The computerized version of WCST was used in the present study [33]. The test consists of four different types of stimulus cards (triangle, star, cross and circle). Participants are given a set of target cards and requested to detect sorting principles (form, color and number) and to match each target card with one of the four stimulus cards. However, the sorting pattern changes after 10 sequential correct responses and participants must switch to a new sorting pattern based on the feedback (correct or incorrect). After 128 trials or when participants achieved nine reversals, the task ends. The total sum of wrong responses, the total sum of perseverative responses, the total sum of perseverative errors are calculated for analyses.

### Statistical analysis

SPSS 16.0 was used to perform the statistical analyses. We tested for effects of gender on TA scores, decision making performances, TMT and WCST scores using one-way analysis of variance (ANOVA). Furthermore, we tested for effects of TA on IGT performance using a repeated measures analysis of variance (ANOVA) with block as the within-subjects factor and TA group as the between-subjects factor. A one-way ANOVA with block as the between-subjects factor was performed to examine the influence of decision process on the IGT netscore, and a one-way ANOVA with group as the between-subjects factor was performed to examine individual deck level preference. The effects of TA on GDT performance were also tested using a repeated measures ANOVA with choice as the within-subjects factor and TA group as the between-subjects factor. The effects of choice were analyzed using a one-way ANOVA with TA group as factor. The threshold of statistical significance was set at *p* < .05.

## Results

### Overall TA score

Within our sample, the TA scores ranged from 20 to 63, with a mean score of 38.15 (*SD* = 7.84). We did not find a statistically significant difference between men and women on the TA scores (*t*(1,302) = −0.23, *p* = .816). Inclusion criteria for a high trait anxiety (HTA) group and low trait anxiety group (LTA) criteria were set at ± 1 SD (7.84) of the mean. Thus, those scoring 30 or below (47 males and 41 females) or 46 and above (52 males and 45 females) met criteria for the LTA and HTA groups, respectively. Participants scoring between 37 and 39 inclusively composed our narrow medium trait anxiety (MTA) group (62 males and 57 females). The use of TA scores around the mean for the selection of MTA group led to a clear separation of groups. This categorization method was also used in the study by Miu et al. [10] and the study by de Visser et al. [3]. There were significant differences between the TA scores of the three groups (HTA, 51.68 ± 4.16; MTA, 37.84 ± 0.80; LTA, 26.23 ± 2.75, F(2,301) = 1892, p < .001). We found no significant differences between men and women on the TA score in any group (HTA, *t*(1,95) = −1.05, *p* = .298; MTA, *t*(1,117) = 0.43, *p* = .666; LTA, *t*(1,86) = −0.44, *p* = .661). The sex proportion within the three groups had no marked difference (^2^ = 0.06, *p* = .971).

### Decision making on the IGT

#### Netscore on the IGT

**Fig 1:**
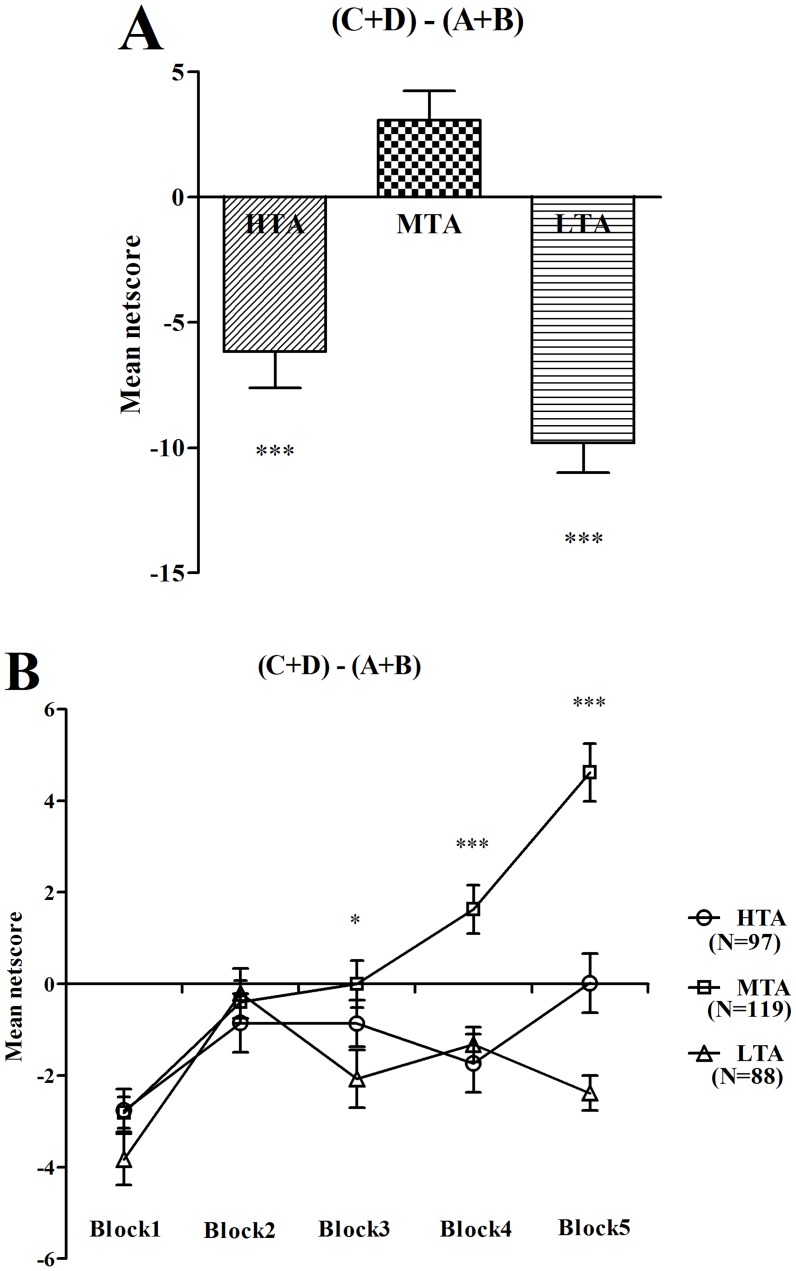
Netscore over the task and netscore of the five blocks during the IGT. Mean netscore over 100 picks of cards (A) and mean netscore for each block of 20 trials (B) for subjects with HTA, MTA, and LTA. *p < .05 and ***p < .001. Means ± SEMs are shown.

A one-way ANOVA with group as the between-subjects factor was performed to examine the IGT netscores. There were significant differences between the IGT netscores of the three groups (*F*(2,301) = 27.95, *p* < .001). The MTA group scored higher than the HTA and the LTA groups (all *p*s < .001), and there was no significant differences between the HTA and LTA groups (*p* = .058) (Fig 1A). To examine the IGT performances in more detail, a repeated-measures ANOVA with block as the within-subjects factor and group as the between-subjects factor was performed. There was a significant main effect for group (*F*(2,301) = 27.95, *p* < .001), indicating that the MTA group performed better than the HTA and the LTA groups, for block (*F*(4,1204) = 21.76, *p* < .001), indicating that IGT performance changed significantly over time, and for group by block interaction (*F*(4,1204) = 8.24, *p* = .001). Comparisons of two of the groups revealed different patterns of performance between the MTA and the HTA groups (group effect: *F*(1,214) = 24.84, *p* < .001; block effect: *F*(4,856) = 23.50, *p* < .001; group by block interaction: *F*(4,856) = 7.28, *p* < .001), between the MTA and the LTA groups (group effect: *F*(1,205) = 55.62, *p* < .001; block effect: *F*(4,820) = 21.48, *p* < .001; group by block interaction: *F*(4,820) = 14.41, *p* < .001), and between the HTA and the LTA groups (group effect: *F*(1,183) = 3.68, *p* = .057; block effect: *F*(4,732) = 7.30, *p* < .001; group by block interaction: *F*(4,732) = 2.72, *p* = .029).

In the IGT, the change curve of netscore indicates the changes in decision strategies. A one-way ANOVA with block as the between-subjects factor was performed to examine the influence of decision process on the IGT netscore. Decision process had significant influence on the netscore of the MTA group (*F*(4,590) = 29.67, *p* < .001). Decision process had significant effect on the netscore of the HTA group (*F*(4,480) = 3.30, *p* = .011). Also decision process had significant effect on the netscore of the LTA group (*F*(4,435) = 6.95, *p* < .001). Single comparisons of performances on the five blocks between groups indicated significant netscore differences in block 3 (*F*(2,301) = 3.57, *p* = .029), block 4 (*F*(2,301) = 12.61, *p* < .001) and block 5 (*F*(2,301) = 38.01, *p* < .001) (Fig 1B). In addition, we did not find statistically significant differences between men and women on the IGT netscore (*t*(1,302) = −0.39, *p* = .697). To further examine the gender differences, a repeated-measures ANOVA with block as the within-subjects factor and gender as the between-subjects factor was performed. There was no significant main effect for gender (*F*(1,302) = 0.14, *p* = .713) or a group by block interaction (*F*(4,1208) = 0.03, *p* = .861). There was a significant main effect for block (*F*(4,1208) = 15.65, *p* < .001), indicating that IGT performance changed significantly over time.

#### Individual deck level preference

**Fig 2:**
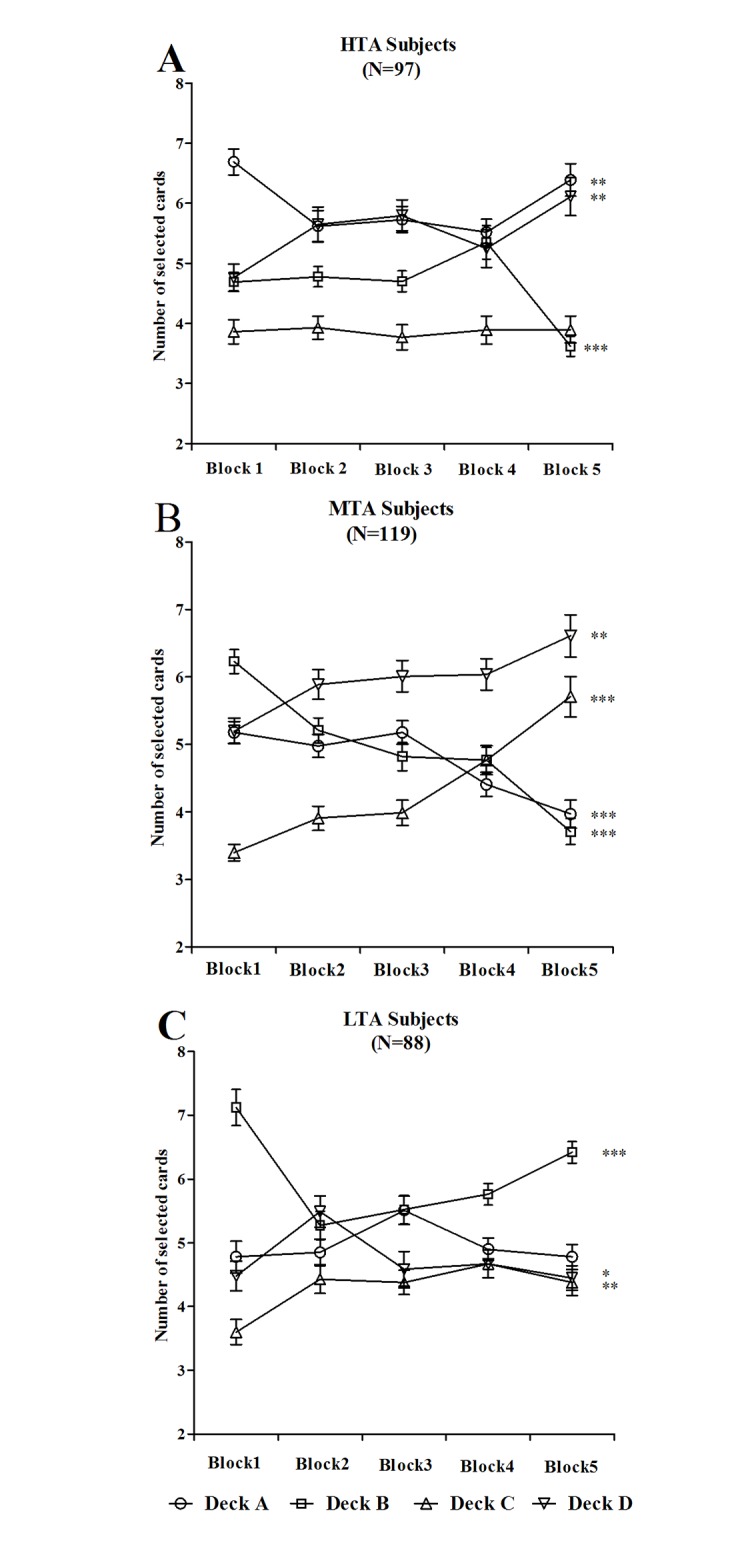
Number of cards selected in blocks during the IGT. Mean number of cards selected from individual deck A, B, C and D for subjects with HTA (A), MTA (B) and LTA (C), graphed as a function of trial block. MTA subjects gradually increased their preference for advantageous decks (C and D) and decreased their preference for disadvantageous decks (A and B). *p < .05, **p < .01 and ***p < .001. Means ± SEMs are shown.

A one-way ANOVA with block as the between-subjects factor was performed to examine the number of cards selected in individual deck A, B, C and D in each block on the IGT. In the HTA group, the number of cards selected changed significantly over the course of the task in decks A (*F*(4,480) *=* 4.80, *p* = .001), B (*F*(4,480) *=* 10.75, *p* < .001) and D (*F*(4,480) *=* 3.40, *p* = .009), but not in deck C (*F*(4,480) = 0.08, *p* = .989) (Fig 2A). In the MTA group, the number of cards selected changed significantly over the course of the task in decks A (*F*(4,590) *=* 8.89, *p* < .001), B (*F*(4,590) *=* 22.27, *p* < .001), C (*F*(4,590) = 18.65, *p* < .001) and D (*F*(4,590) *=* 4.35, *p* = .002) (Fig 2B). In the LTA group, the number of cards selected changed significantly over the course of the task in decks B (*F*(4,435) = 11.89, *p* < .001), C (*F*(4,435) *=* 3.77, *p* = .005) and D (*F*(4,435) *=* 3.35, *p =* .01), but not in deck A (*F*(4,435) *=* 2.15, *p* = .074) (Fig 2C).

**Fig 3:**
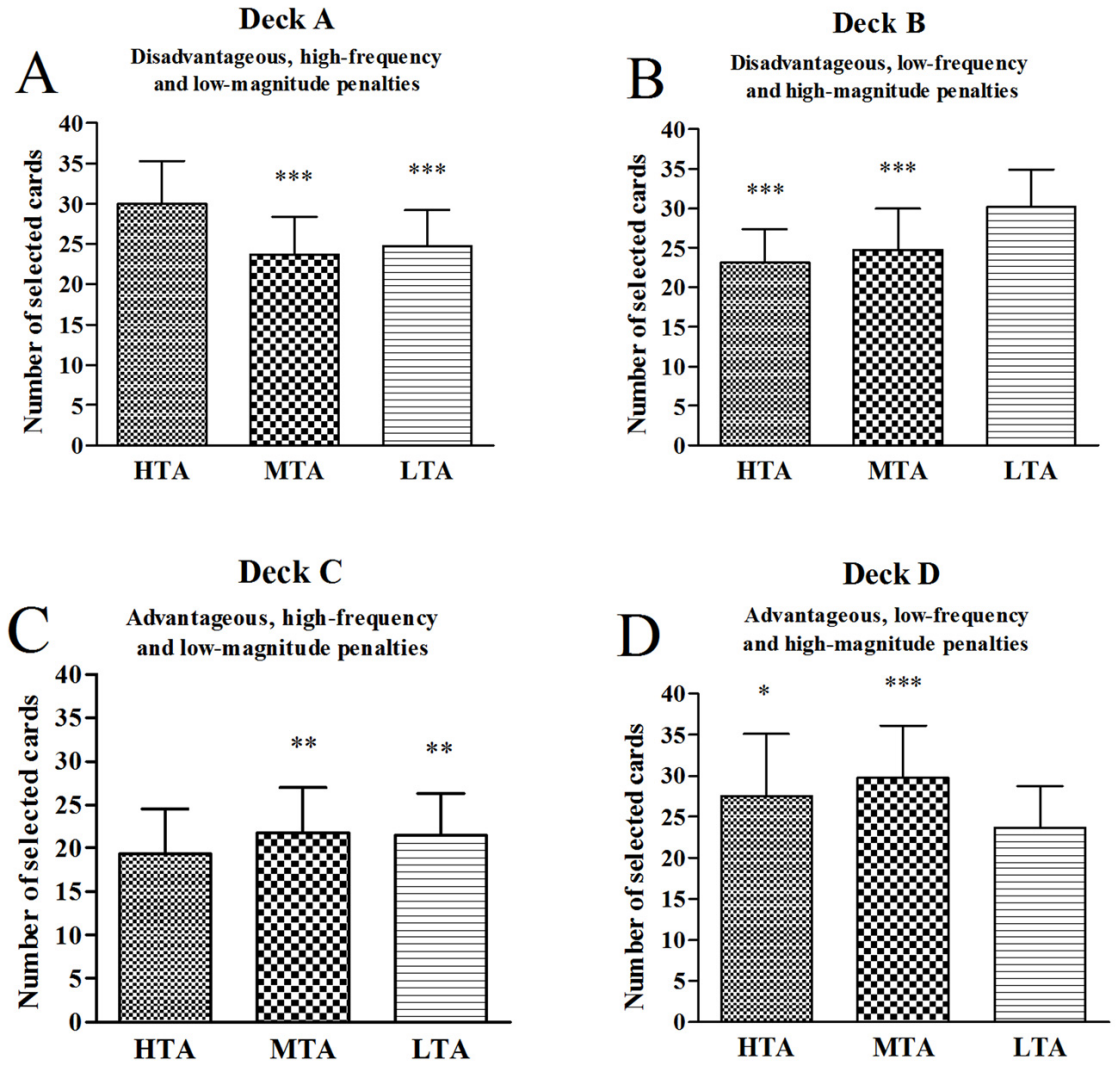
Number of cards selected for groups during the IGT. Mean number of cards selected for subjects with HTA, MTA, and LTA from individual decks A (A), B (B), C (C), and D (D) over 100 picks of cards. *p < .05, **p < .01 and ***p < .001. Means ± SEMs are shown.

A one-way ANOVA with group as the between-subjects factor was performed to examine individual deck level preference. There were significant differences in deck A overall score between the three groups (*F*(2,301) = 17.95, *p <* .001), and post hoc LSD test revealed that the HTA group selected significantly more cards from the deck A than the MTA and LTA groups did (all *p*s *<* .001), with no significant differences between the MTA and LTA groups (*p =* .098) (Fig 3A). There were significant differences in deck B overall score between the three groups (*F*(2,301) = 30.19, *p <* .001), and post hoc LSD test revealed that the LTA group selected significantly more cards from the deck B than the MTA and HTA groups did (all *p*s *<* .001), with no significant differences between the MTA and HTA groups (*p =* .234) (Fig 3B). There were significant differences in deck C overall score between the three groups (*F*(2,301) = 6.90, *p =* .001), and post hoc LSD test revealed that the HTA group selected significantly less cards from the deck C than the MTA and LTA groups did (all *p*s *<* .01), with no significant differences between the MTA and the LTA groups (*p =* .647) (Fig 3C). There were significant differences in deck D overall score between the three groups (*F*(2,301) = 22.91, *p <* .001), and post hoc LSD test revealed that the LTA group selected significantly less cards from the deck D than the HTA and MTA groups did (all *p*s *<* .001), with significant differences between the HTA and MTA groups (*p =* .014) (Fig 3D).

**Fig 4:**
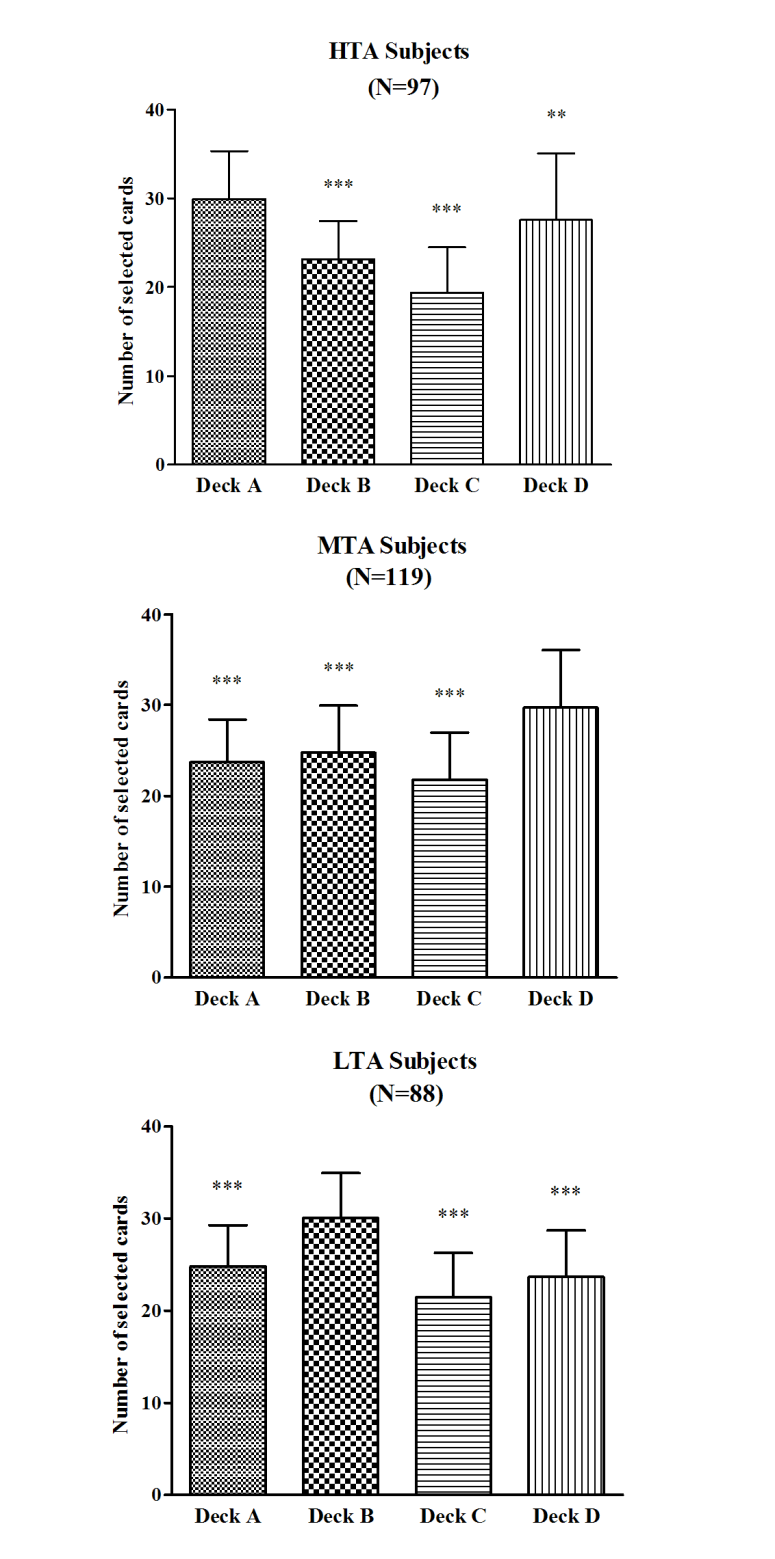
Number of deck selections during the IGT. Mean number of selections for individual decks A, B, C, and D in the HTA (A), MTA (B), and LTA (C) subjects over 100 picks of cards. *p < .05, **p < .01 and ***p < .001. Means ± SEMs are shown.

A one-way ANOVA with deck as the between-subjects factor was performed to examine individual deck level preference for each group. In the HTA group, the number of cards selected differed between the four decks (*F*(3,384) = 66.38, *p <* .001), and post hoc LSD test revealed that the HTA group selected significantly more cards from the deck A than from the decks B (*p <* .001), C (*p <* .001) and D (*p =* .004) (Fig 4A). In the MTA group, the number of cards selected differed between the four decks (*F*(3,472) = 47.07, *p <* .001), and post hoc LSD test revealed that the MTA group selected significantly more cards from the deck D than from the decks A, B and C (all *p*s *<* .001) (Fig 4B). In the LTA group, the number of cards selected differed between the four decks (*F*(3,348) = 51.79, *p <* .001), and post hoc LSD test revealed that the LTA group selected significantly more cards from the deck B than from the decks A, C and D (all *p*s *<* .001) (Fig 4C).

### Decision making on the GDT

**Fig 5:**
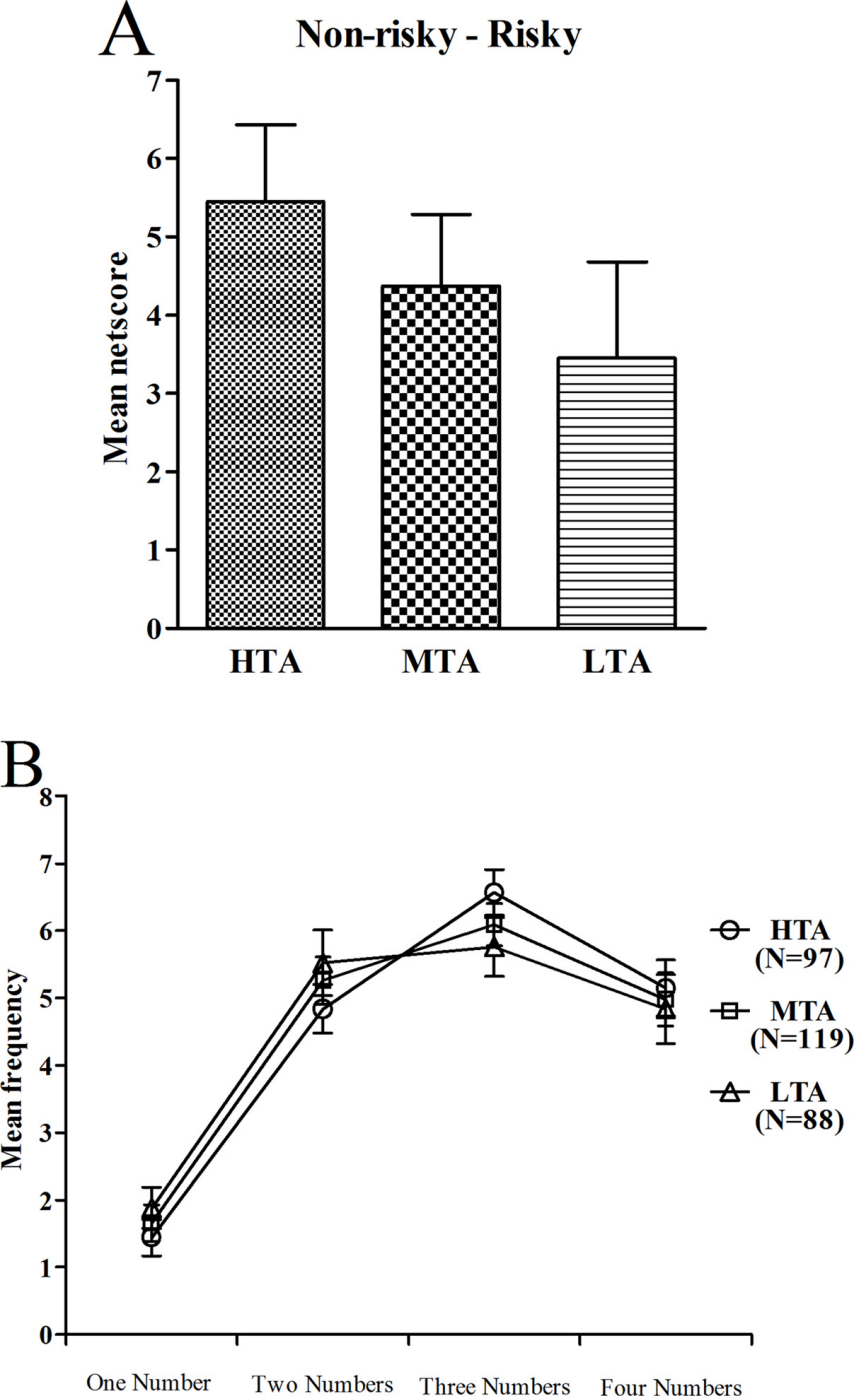
Netscore and frequency of each single alternative during the GDT. Mean netscore over the GDT (A) and mean frequency of each single alternative (B) for subjects with HTA, MTA, and LTA. Means ± SEMs are shown.

A one-way ANOVA with group as the between-subjects factor was performed to examine the GDT netscores. In contrast to the IGT, there was no significant difference between the netscores of the three groups (*F*(2,301) = 0.87, *p* = .420) (Fig 5A). Moreover, we did not find statistically significant differences between men and women on the GDT netscore (*t*(1,302) = 0.62, *p* = .538). An ANOVA with repeated measures with choice as the within-subjects factor and group as the between-subjects factor was conducted. There was a significant main effect for choice (*F*(3,903) = 61.37, *p* < .001), but no significant main effect for group (*F*(2,301) = 1.07, *p* = .345) and no significant interaction between choice and group (*F*(3,903) = 0.59, *p* = .735), which indicated that there were no differences between the three groups on the GDT. None of the single comparisons for the different choices reached significance between groups: one number, *F*(2,301) = 0.57, *p* = .569; two numbers, *F*(2,301) = 0.71, *p* = .491; three numbers, *F*(2,301) = 1.18, *p* = .308; four numbers, *F*(2,301) = 0.12, *p* = .889 (Fig 5B). Furthermore, there was no correlation between the GDT netscore and the IGT netscore as well as between the GDT netscore and the score in each block in the three groups (all *p*s *>* .05).

We examined the use of negative feedback (losses) after the decision of a risky option to choose a non-risky option in the next trial; only those participants who chose a risky option and received negative feedback at least once during the GDT were included. Thus, the data of 263 subjects were analyzed. The three groups did not differ on the use of negative feedback (HTA: n = 83, *M* = 55.06%, *SD* = 39.26; MTA: n = 103, *M* = 50.74%, *SD* = 38.79; LTA: n = 77, *M* = 52.99%, *SD* = 39.89, *F*(2,260) = 0.28, *p* = .756). The feedback use was significantly associated with the GDT netscore (HTA: *r* = 0.77, *p* < .001; MTA: *r* = 0.68, *p* < .001; LTA: *r* = 0.49, *p* < .001). We also examined the use of positive feedback (gains) after the decision of a non-risky option to choose a non-risky option again; only those participants who chose a non-risky option and received positive feedback at least once during the GDT were included. Thus, the analysis was based on the data of 290 participants. There was no significant differences between the three groups with regard to the use of positive feedback (HTA: n = 96, *M* = 61.63%, *SD* = 33.70; MTA: n = 112, *M* = 58.54%, *SD* = 34.51; LTA: n = 82, *M* = 63.60%, *SD* = 33.01, *F*(2,287) = 0.55, *p* = .575). The use of positive feedback was also significantly associated with the GDT netscore (HTA: *r* = 0.79, *p* < .001; MTA: *r* = 0.68, *p* < .001; LTA: *r* = 0.33, *p* = .002). There were no significant group differences on feedback utilization (all *F*s < 1.67, all *p*s > .177).

### Neuropsychological background testing

We did not find any statistically significant differences between the three groups on the TMT and the WCST (all *p*s > .218) (see Table 1 for M and SD). Thus, trait anxiety did not influence performances on the TMT and the WCST. And we did not find statistically significant differences between men and women on the TMT and the WCST (all *p*s > .177). In the three groups, the GDT netscore were correlated with wrong responses (HTA: *r* = −0.36, *p* < .001; MTA: *r* = −0.24, *p* = .009; LTA: *r* = −0.36, *p* = .001) and perseverative errors (HTA: *r* = −0.29, *p =* .005; MTA: *r* = −0.24, *p* = .008; LTA: *r* = −0.32, *p* = .003). However, there was no correlation between all the neuropsychological indexes and the IGT netscore as well as between all the neuropsychological indexes and the score in each block in the three groups (all *p*s > .05).

**Table 1:** Results of the neuropsychological tests in the three groups [mean (SD)].

	HTA (n = 97)	MTA (n = 119)	LTA (n = 88)	*F*	*p*
TMT					
TMT A (s)	24.66 (2.74)	25.39 (3.24)	25.21 (3.61)	1.44	0.238
TMT B (s)	50.22 (5.30)	49.42 (5.26)	49.13 (4.48)	1.18	0.309
WCST					
Wrong responses	49.14 (23.83)	44.52 (22.53)	49.31 (24.07)	1.46	0.233
Perseverative response	58.14 (29.57)	53.11 (27.96)	59.23 (29.93)	1.35	0.260
Perseverative errors	33.23 (21.88)	28.82 (18.96)	32.69 (20.47)	1.53	0.219

Note: TMT: Trail Making Test; WCST: Wisconsin Card Sorting Test; HTA: high trait anxiety; MTA: medium trait anxiety; LTA: low trait anxiety.

## Discussion

We investigated the interaction between trait anxiety and complex decision making. The present study yielded two main results. The primary finding was that trait anxiety had an effect on decision making under ambiguity but not decision making under risk in healthy participants with different levels of TA. While the HTA and LTA subjects performed worse on the IGT compared to the MTA subjects, performances on the GDT between the three groups did not differ. Furthermore, the LTA and HTA groups showed different individual deck level preferences on the IGT: the former had a preference for deck B indicating that these subjects focused more on the magnitude of rewards, and the latter showed a preference for deck A indicating significant decision making impairment. In general, we replicated the study which examined the relationship between IGT and TA and extended the research content by examining the association between GDT and TA. In addition, we analyzed individual deck level preferences on the IGT for the first time.

The LTA subjects performed worse on the IGT. They seemed attracted to immediate gains and were unable to develop a profitable long-term strategy quickly. This result is agreement with the somatic marker hypothesis (SMH), which offers a cognitive framework for decision making and a systematic and paradigmatic explanation of how emotions affect decision making [42]. The key feature of the SMH is that emotion plays an important role in decision making, and emotion-related signals (somatic markers) generated by the body, such as skin conductance responses (SCRs) and heart rate, help individuals regulate decision making, particularly under circumstances of uncertainty and complexity. Thus, when individuals make decisions, specific somatic markers are generated and stored in memory for every response option and related emotional reaction. That is to say, a connection between each response option and the somatic markers associated with a specific emotional state is founded when individuals make decisions and influences subsequent selections. With respect to the intensity of the connection, people with a strong or weak disposition toward the experience of emotional states may differ [11]. Therefore, individuals disposed to weak emotional states, such as our LTA subjects, may develop inferior somatic markers and perform poorly on a decision making test. This is also supported by the findings of Werner et al. [11] who reported a positive correlation between trait anxiety and IGT performance. Furthermore, previous studies have found that the vmPFC/OFC and amygdala are crucial regions underlying somatic state activation [43]; low anxiety has been associated with decreased amygdala and vmPFC activation [44,45]. Thus, we could speculate that failure to fully activate the somatic marker system results in impaired IGT performance in our LTA subjects, in line with the SMH that somatic markers play an important role in guiding profitable decision making.

There is another interpretation of the failure of our LTA subjects to perform poor on the IGT. This interpretation hinges on the fact that the IGT has been regarded as a measurement of “contingency learning”, and contingency learning ability is related to performance on the IGT [46,47]. The OFC, in particular, has been frequently implicated in contingency learning [48]. Human imaging studies have found that the OFC is involved in relearning and re-evaluating contingencies [49]. Many human lesion studies have shown that lesions to the OFC are related to a deficit in the relearning of contingencies. Such lesions are associated with alterations in the learning of associations between choice selections and reward outcomes, manifested chiefly by perseverative behavior to previously rewarded stimuli following the reversal of reinforcement contingencies [50]. Similarly, our LTA subjects have been shown to have decreased activation within the OFC [44,45], therefore, it is not difficult to understand their repeated choice of the disadvantageous decks in the IGT in the current study.

The HTA subjects also had a poor IGT performance. The result seems to contradict the SMH in that individuals with a disposition to experience strong emotional states (our HTA subjects) may perform better on the IGT. Nevertheless, the result is accordance with the study by de Visser et al. [3], who reported that healthy subjects with high TA had poorer performance on the IGT. More interestingly, highly anxious rats had poor performances on the rat analogue of the IGT [51]. Bechara et al. [43] suggested that some individuals, such as HTA individuals, might abolish the adaptive influence of the somatic signals by means of higher cognitive processes. Several candidate mechanisms could account for poor performance in HTA individuals. One of the most widely accepted, commonly repeated theories is attentional control theory [52]. The ACT was put forward to account for the detrimental influence of anxiety on cognitive functions, and highlights that anxiety as a personality trait is closely related to individual differences in higher-order functions of cognitive control. Central to the predictions of the ACT was the assumption that anxiety influences inhibition and shifting functions. Inhibition involves attentional control to resist interference from distracting and task-irrelevant stimuli, and shifting involves attentional control to maintain focus on changing task demands [52]. Specifically, HTA is thought to bias the balance between a goal-driven attentional system and a stimulus-driven attentional system in favor of the latter [53]. Consequently, task-irrelevant information cannot be fully inhibited and should be more intrusive in highly anxious individuals, resulting in impaired cognitive task performance [52,54]. The ACT also argues that anxiety may result in interference from excessive declarative elaboration (rumination), which HTA individuals may place on each alternative in the IGT [6], but such declarative elaboration is useless in the first half of the IGT. The ACT has suggested that shifting is disrupted in anxious individuals [52]. In the IGT, shifting is defined as the ability to switch attention between the four decks and to constantly update and monitor representations of each deck. A similar assumption has been put forward to expound the effect of trait anxiety on cognitive functions. In the dual mechanisms of control account, Braver et al. [55] proposed a link between individual differences in trait anxiety and the general manner in which cognitive control is exerted. During a complex task, highly anxious individuals may engage control in a reactive and passive way, not a sustained and proactive way. Consequently, bottom-up input and goal-irrelevant information will be more influential [56].

Another important factor potentially contributing to inconsistent findings in the relationship between TA and IGT performance is individual deck level preferences in the IGT. The role of individual deck level preferences in assessing IGT performance were overlooked by most of the previous studies [57]. Although decks A and B lead to long-term negative outcomes, deck A includes high-frequency and low-magnitude rewards but deck B includes low-frequency and high-magnitude rewards. Greater deck A or greater deck B selections depend on whether subjects focus on the frequency of the rewards or the magnitude of rewards. The IGT manual demonstrates that, while avoiding deck B is considered a good decision, deck A should be avoided by most “neurologically intact” individuals [41]. In the current study, the HTA group selected significantly more cards from deck A than the MTA and LTA groups did, and selected significantly more cards from deck A than from decks B, C and D; the LTA group selected significantly more cards from deck B than the HTA and MTA groups did, and selected significantly more cards from deck B than from decks A, C and D. This result demonstrates that while the LTA subjects showed a preference for deck B indicating that these subjects focus more on the magnitude of rewards, the HTA subjects showed a preference for deck A indicating significant decision making impairment. This result is in accordance with the study by Buelow and Suhr [57] who reported personality traits related differently to selecting deck A versus deck B.

Our results provide some interesting implications for the roles of different levels of TA in influencing individual deck selections on the IGT, and emphasize the importance of examining selections from individual decks separately as there were differences in the number of selections of decks A and B between the HTA and LTA subjects in our study. Such a measurement would allow for consideration of whether subjects who are impaired on the IGT (i.e., selecting more disadvantageous than advantageous choices) prefer deck A, the deck more sensitive to impaired decision making (“pathological” decision making), or instead prefer deck B, a deck sensitive to risky decision making but less sensitive to decision making impairments [58]. Therefore, combining decks A and B into a disadvantageous deck may cover up individual deck level preferences [57].

In summary, the LTA and HTA subjects demonstrated poor performance on the IGT compared with the MTA subjects. Therefore, the relationship between trait anxiety and IGT performance resembles an inverted U-shaped curve. At the beginning of the task, all subjects preferred the decks containing high immediate gains. The MTA subjects seemed to realize that high immediate gains were accompanied by higher losses and gradually turned to the advantageous decks that contained small immediate gains but even smaller losses over the course of the task. However, the HTA and LTA subjects seemed to prefer disadvantageous choices and maintained this behavior pattern even after suffering greater losses. The HTA and LTA subjects tended to achieve high immediate gains at the cost of accepting higher losses, and they abided by the strategy of giving priority to immediate gains. This is in accordance with the ‘myopia for the future’—foregoing long-term benefit for short-term profit—proposed by Bechara [59]. On the contrary, the MTA subjects first considered avoiding losses and then considered the high gains; therefore, they followed a strategy of giving priority to safety.

Contrary to the IGT, there were no performance differences on the GDT between subjects with different levels of TA. Brand and colleagues [13] have suggested that there may be two interacting routes that can guide decision making under risk as measured by the GDT: a cognitive route, in which information about consequences and probabilities are integrated and utilized before a decision is made, and an emotional route, in which feedback in terms of rewards and punishments is processed. In accordance with the proposed cognitive route, previous studies with a variety of patient groups and healthy subjects have found a relationship between impaired GDT performance and executive dysfunctions, such as set-shifting, cognitive flexibility and categorization as measured by the WCST [24,60]. Meanwhile, other studies found that subjects with intact executive functions were unimpaired on the GDT [61,62]. Additionally, in a particularly noteworthy study with three Urbach-Wiethe patients, while the two patients with reduced executive functioning showed impaired GDT performance, the other one with unimpaired performance across all executive tasks performed normally on the GDT [16]. We suggest that this association is most likely due to participants using information about the contingencies of the GDT that are explicitly presented to plan, monitor and modify profitable strategies. Based on these findings, we inferred that intact executive functions are a main component in unimpaired GDT performance. In line with this inference, there were no significant differences in executive functions, as measured by the WCST and TMT, between our subjects with different levels of TA.

In agreement with the assumed emotional route, decision making performance on the GDT was correlated with processing feedback in terms of gains and losses. In a previous study, participants suffering from binge eating disorder selected the disadvantageous options significantly more than the control group did. With regard to capacities to advantageously use feedback processing, subjects with binge eating disorder stayed with safe decision less often than controls in response to positive feedback after a safe decision. Meanwhile, they also changed their strategy after negative feedback on a risky decision less often than the controls [63]. Similar results were found in another study with patients with ADHD [32]. Therefore, we could hypothesize that deficits in the GDT are related to disadvantageous utilization of feedback from previous choices. However, in our study, the three groups of subjects had no significant difference in processing feedback. They used a loss (negative feedback) after a risky decision to choose a safe option and used a gain (positive feedback) after a non-risky decision to choose a safe option again. Moreover, Labudda et al. [61] suggested that feedback processing in the GDT is not particularly necessary for determining advantageous decisions when executive functions are unimpaired, but it is an additional route of information.

It should be noted that the mean TA scores of the three groups were similar to scores obtained with similar categorization methods [3] and with different categorization methods (quartiles) [64]. Moreover, the mean TA scores of our samples are comparable to the TA scores of average Chinese college students. This indicates that the sample of the present study represents the distribution of TA in the general Chinese population. In regard to trait anxiety, some studies have found that women tend to show higher overall levels of trait anxiety compared to men [3]. However, there are also many studies showing that women do not differ from men with regard to trait anxiety [65,66]. Cultural differences may partly account for the differences between the same types of studies. As a case in point, it is worth mentioning some cross-cultural studies on neuroticism as van den Bos et al. have stated [12]. Neuroticism has been related to trait anxiety [67], and has been shown to be higher in females compared to males across most nations. More significantly, sex differences in neuroticism were more remarkable in nations with high levels of development than in nations with low levels of development [68,69]. Researchers have proposed that sex differences under less constrained conditions are more remarkable than under more constrained conditions [68]. Thus, cross-cultural studies are certainly needed to further reveal the essence of these intriguing differences in trait anxiety.

In the present study, we did not find any differences on IGT performance between males and females. In regards to sex differences, the literature is contradictory in terms of performance differences. Some studies did not find sex-related differences in IGT performance [10,38], whereas some other studies have found that men choose more advantageously and out-perform women on the IGT [70,71,72,73]. Unfortunately, many of those studies did not employ the original version of the 100 IGT trials, but instead used 120 IGT trials [70], 150 IGT trials [71] or 200 IGT trials [72,73]. These differences render a direct comparison with our data unfeasible. Another important reason could be the way we have classified our participants. In the current study, decision making performance and sex differences on decision making performance were compared based on the chosen participants, but not all participants who were initially enrolled.

With respect to the effect of depression on decision making, previous studies have found that depressed individuals show impaired decision making in static and dynamic environments and tend to make less advantageous choices on the IGT [74,75]. Altered sensitivity to reward and punishment [74], more exploratory behavior in their decision making [76], and attenuated processing of counterfactual outcomes [77] may account for the poor decision making of individuals with depression. Our participants had no current depression or history of depression, which ruled out any effect of depression in the results.

There is also a issue worth exploring. Some studies have indicated that the IGT and GDT share some basic components. They have found that the IGT becomes more explicit in the later blocks and the mechanisms that underlie the IGT are similar to those in the GDT [62,78]. These results showed that there might be a shift from implicit to explicit knowledge for IGT contingencies. Therefore, it is necessary to investigate the time when the shifting emerges and the potential effect of conscious knowledge of contingencies on decision making in future studies.

A limitation of the current study should be addressed. Successful performance on the IGT has been suggested to depend on emotional processing supported by physiological measurement during task performance [79]. The issue of whether the IGT performance in our study is regulated by emotions should be investigated in further studies measuring subjects’ emotional reactivity during the task (through skin conductance response, heart rate, or pupil dilation).

In conclusion, our study demonstrates that trait anxiety had effect on decision making under ambiguity but not decision making under risk. The results provide support for the idea that emotions influence human cognitive functions. Moreover, they extend the view that trait anxiety has impact on decision making. Further work is required to confirm our findings and to elucidate the mechanisms involved by using different methodologies, such as event-related potentials, functional magnetic resonance imaging, and resting-state functional connectivity. Ultimately, the aim is to uncover the science of the interactions between emotion and decision making, as well as other cognitive functions.

## Supporting Information

S1 Dataset TA scores, IGT netscores and GDT netscores for all participants (SPSS). (RAR)
